# Executable pathway analysis using ensemble discrete-state modeling for large-scale data

**DOI:** 10.1371/journal.pcbi.1007317

**Published:** 2019-09-03

**Authors:** Rohith Palli, Mukta G. Palshikar, Juilee Thakar

**Affiliations:** 1 Medical Scientist Training Program, University of Rochester, Rochester, New York, United States of America; 2 Biophysics, Structural, and Computational Biology Program, University of Rochester, Rochester, New York, United States of America; 3 Department of Microbiology and Immunology, University of Rochester, Rochester, New York, United States of America; 4 Department of Biostatistics and Computational Biology, University of Rochester, Rochester, New York, United States of America; Institute for Systems Biology, UNITED STATES

## Abstract

Pathway analysis is widely used to gain mechanistic insights from high-throughput omics data. However, most existing methods do not consider signal integration represented by pathway topology, resulting in enrichment of convergent pathways when downstream genes are modulated. Incorporation of signal flow and integration in pathway analysis could rank the pathways based on modulation in key regulatory genes. This implementation can be facilitated for large-scale data by discrete state network modeling due to simplicity in parameterization. Here, we model cellular heterogeneity using discrete state dynamics and measure pathway activities in cross-sectional data. We introduce a new algorithm, Boolean Omics Network Invariant-Time Analysis (BONITA), for signal propagation, signal integration, and pathway analysis. Our signal propagation approach models heterogeneity in transcriptomic data as arising from intercellular heterogeneity rather than intracellular stochasticity, and propagates binary signals repeatedly across networks. Logic rules defining signal integration are inferred by genetic algorithm and are refined by local search. The rules determine the impact of each node in a pathway, which is used to score the probability of the pathway’s modulation by chance. We have comprehensively tested BONITA for application to transcriptomics data from translational studies. Comparison with state-of-the-art pathway analysis methods shows that BONITA has higher sensitivity at lower levels of source node modulation and similar sensitivity at higher levels of source node modulation. Application of BONITA pathway analysis to previously validated RNA-sequencing studies identifies additional relevant pathways in *in-vitro* human cell line experiments and *in-vivo* infant studies. Additionally, BONITA successfully detected modulation of disease specific pathways when comparing relevant RNA-sequencing data with healthy controls. Most interestingly, the two highest impact score nodes identified by BONITA included known drug targets. Thus, BONITA is a powerful approach to prioritize not only pathways but also specific mechanistic role of genes compared to existing methods. BONITA is available at: https://github.com/thakar-lab/BONITA.

## Author summary

21st-century biotechnology has enabled measurements of genes and proteins at large scale by RNA sequencing and proteomics technologies. In particular, RNA-sequencing has become a first step of unbiased interrogation. These studies frequently produce a long list of differentially abundant genes, which become interpretable by widely used pathway analysis methods. The pathway topologies frequently include information on how genes interact and influence each other’s expression, but current methods do not utilize this information to estimate signal flow through each pathway. We have developed a model of binary (on/off) behavior that accounts for varying expression across samples as different proportions of cells expressing genes. We model signal flow by averaging repeated simulations of individual cells passing binary signals through molecular networks. We use this model to infer regulatory rules explaining gene expression. These rules of signal integration for all nodes in the network are used to identify the most important genes, and to determine if a pathway’s activity is different between two groups. BONITA compares favorably to previous approaches using simulated and real data. Furthermore, application to 36 datasets from 15 different diseases demonstrates BONITA’s exceptional ability to detect drug targets.

This is a *PLOS Computational Biology* Methods paper.

## Introduction

Gene set and pathway analysis have become one of the first choices for gaining mechanistic insights from high-throughput sequencing and gene/protein profiling techniques [1]. Typically, gene set analysis uses a set of pathway genes to estimate its modulation and discounts pathway topology. This approach ignores synergy among genes, resulting in enrichment of convergent pathways when downstream genes are modulated. Though none of the existing methods explicitly investigate synergy among genes, current topology-based methods use graph theoretical metrics to weigh pathway nodes based on connectivity before estimating pathway modulation [1–3]. However, it is critical to go beyond this simple characterization in order to identify key regulators from large-scale datasets for systematic prioritization of follow-up experiments. Discrete state network modeling facilitates prioritization of experiments by using simple logic rules such as ‘AND’ or ‘OR’ to explicitly define signal integration, enabling investigation of cross-talk and downstream events as shown in our previous studies. [4–6].

Discrete state network modeling has been used to study high throughput gene and protein profiling data collected across multiple time-points by utilizing two different underlying models of variation [7, 8] in addition to conventional Boolean modeling. Fuzzy models explain variation in the gene expression levels using multiple states, unlike Boolean models that allow only binary (on/off) states. Recently, fuzzy models have been used to study literature-derived prior knowledge networks using a genetic programming algorithm to derive logic rules from time course data by Liu et al. [9]. Probabilistic Boolean Network models assume that variability arises from ambiguity in logic-rules employed rather than in amount of activation [10], making the counterintuitive assumption that cells randomly employ one of multiple different wirings. Many biological insights have resulted from fuzzy network [11, 12] and Probabilistic Boolean Network [13, 14] models, but there remains great potential for improvement in describing variation and improving applicability to cross-sectional datasets. Unlike time course data, cross-sectional data is collected from multiple samples (and possibly conditions) at a single time point providing minimal information about interactions between genes. Indeed, cross-sectional sampling is more feasible in translational studies and algorithms that derive discrete state network models from this data type would have greater applicability in translational research.

Here, we describe BONITA- Boolean Omics Network Invariant-Time Analysis, to capture cellular heterogeneity, a critical source of variability in transcriptomic data. A portion of variance in gene expression stems from heterogeneity in the activation state of cells in addition to variation in expression levels within each cell. This is demonstrated by gene expression in multiple stem cell types [15] [16] and stimulated bone marrow-derived dendritic cells [17]. BONITA is designed specifically to leverage this bimodality in cell-specific gene expression to perform continuous-valued simulations of molecular networks under assumptions of switch-like behavior in each cell. Hence, BONITA network propagation (NP) assumes that the activity of each biomolecule is directly dependent upon the proportion of cells in which that molecule is active or, equivalently, the probability a node is active in an arbitrary cell. The propagation of signals across multiple cells facilitates the application of NP to the cross-sectional data. Since this NP approach should recapitulate steady states in cross-sectional data, BONITA rule determination (RD) finds rules that minimally change activities after NP. These logic rules representing synergy between genes from cross-sectional data are utilized in BONITA pathway analysis (PA). Thus, by capturing integration of signals coming from multiple genes, BONITA uncovers differentially regulated pathways.

BONITA is currently implemented and tested for application to transcriptomics data, but work is under way to apply it to other types of data including proteomics, metabolomics, and phosphoproteomics. BONITA is rigorously tested using simulated data and is applied to publicly available experimental datasets. In addition, a comparison of BONITA-RD to an existing algorithm for time-course data [9] shows comparable performance for cross sectional data, improving applicability to translational studies. Moreover, comparison of BONITA-PA with state-of-the-art pathway analysis methods CAMERA [18] and CLIPPER [3] shows exceptional Receiver Operating Characteristic (ROC) and higher specificity in detecting signaling modulations in validated experimental studies. Finally, when applied to disease specific data from patients vs healthy humans, BONITA impact scores identify known drug targets as key regulators. This suggests that BONITA can be used for drug discovery from large-scale high-throughput datasets.

## Materials and methods

### BONITA network propagation (BONITA-NP) models signal transduction

BONITA network propagation (NP) runs on prior knowledge networks obtained from the Kyoto Encyclopedia of Genes and Genomes (KEGG) using the KEGG API. Activating/inhibiting relationships are inherited from KEGG edge attributes [19]. Edges in KEGG pathways contain edge type annotations; these are exploited to determine activating or inhibitory edges. Hence, all the Boolean functions inferred by BONITA are sign-compatible functions, i.e., they satisfy positive or negative unateness based on the interaction annotation, as described in Zhou et al [20]. We demonstrate in S1 Text and S1 Fig that BONITA-NP infers these sign-compatible functions in an unbiased manner. BONITA-NP assumes that the mRNA-producing cells are proportional to counts obtained from mRNA-sequencing. To obtain the proportion of cells expressing mRNAs, the RNA-seq data is transformed to [0, 1] domain using division by the maximum element. This transformed data is used as a starting point to compute a series of Boolean Network simulations using synchronous or asynchronous update algorithms as described in [21]. The ensemble averages of 1000 such repeated runs are used to define activities which are compared with the transformed data to determine fitness value in BONITA-RD below. Comparison of methods for data transformation to [0, 1] demonstrated that division by maximum was the best method for transforming data and that BONITA-RD, as expected, has better fits than purely Boolean simulation (S2 Text, S2 Fig). In this report, all BONITA-NP simulations were carried out for 100 steps using the synchronous update algorithm. The maximum number of steps necessary to reach the steady state or terminal cycle of the Boolean network is the longest path between any two nodes in the network. The longest shortest path between all pairs of nodes across KEGG networks was 17 (S3 Text, S3 Fig) indicating that 100 simulation steps were adequate. The results were reported as average over the last ten steps of the simulation.

### BONITA-RD algorithm for rule determination

BONITA-RD implements a combination of a genetic algorithm and a node-wise local search to infer logic-rules. BONITA assumes cross-sectional samples represent steady states and minimizes change after simulation of a network as given by:

$$\sum_{i=1}^{d}\frac{1}{n}\sum_{j=1}^{n}{\left(D_{i,j}-O_{i,j}\right)}^{2}$$ (1)

**Fig 1:**
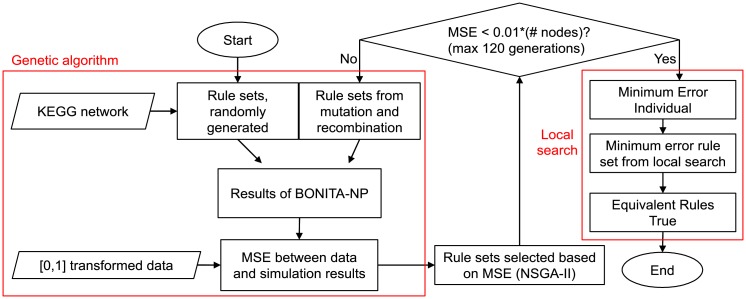
BONITA rule determination (BONITA-RD) flowchart shows dependencies between BONITA-NP, genetic algorithm and local search for rule inference. Rhomboids represent inputs while rectangles represent calculation steps. MSE is mean square error given by Eq 2. BONITA-NP is BONITA Network Propagation.

In Eq 1, *d* is the number of available samples, *n* is the number of nodes in the network, *D*_*i*,*j*_ is the value of node *j* in sample *i*, and *O*_*i*,*j*_ is the value of node *j* in sample *i* calculated by BONITA-NP. The overall design of the rule determination algorithm is graphically represented in Fig 1.

**Fig 2:**
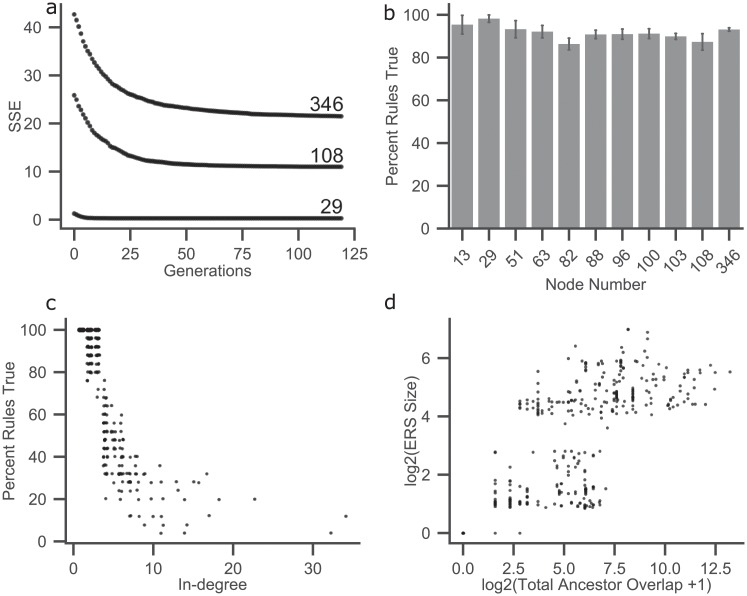
BONITA-RD accuracy and node characteristics. a) Sum of squares of node-wise error (SSE) are plotted across genetic algorithm generations for three representative test networks with varying complexity (node degree = 346, 108, and 29). b) Percent rules true (y-axis) identified by BONITA-RD are plotted against the number of nodes in 11 networks (x-axis) for 25 simulated trials. Error bars represent standard error. c) The percent rules true in ERS (y-axis) for each node are plotted against the in-degree of those nodes in the prior knowledge network. d) The log average total size of ERS for each node are plotted against the log total ancestor overlap which is the sum of the pairwise shared ancestor number between any two upstream nodes.

The genetic algorithm generates new rule sets (individuals) either by selecting rules for randomly chosen nodes from their parent rule sets, or by mutating (altering) a particular rule and incoming nodes. At later generations, crossover events tend to produce rule sets that have already been tried in earlier generations, leading to a greater probability of mutations. The space of potential rules is extremely large and scales quickly with in-degree. Hence, to reduce the space of potential rules to a region that can be sampled, a maximum of three upstream regulators are selected. This is a compromise between decreasing resolution and increasing search time. The three upstream regulators (U) are sampled for nodes with >3 upstream regulators in the genetic algorithm using a probability function $P(U)=\frac{C_{U,N}}{\sum_{U}C_{U,N}}$ where *C*_*U*,*N*_ is the Spearman correlation of upstream regulators with the node (N) for which the rule is being determined. For all simulations shown in this report, the genetic algorithm was run for 120 generations from a starting population and constant population size of 24. Thus, 24 new rule sets were generated and tested at each generation. Decreasing errors (Fig 2a) with a plateau before 40 generations for networks with varying complexities indicated that 120 generations are appropriate for the genetic algorithm. The genetic algorithm searches the product of the number of possible rules at each node in the network. In order to transform this multiplicative problem into an additive one, a node-level local search strategy was implemented. The local search only considers the error at the node under consideration as given by

$$\sum_{i=1}^{d}(D_{i,j}-O_{i,j}{)}^{2}$$ (2)

This exhaustive search only evaluates the possible rules at each node while holding constant all other rules as well as the incoming edges to that node as determined by the genetic algorithm. The node-level local search was initiated with the minimal error rule set from the genetic algorithm and was found to be effective in inferring the rules as shown in the results (Fig 2b). During the local search, rules within a tolerance threshold of this minimal rule are kept as equivalent rules i.e. the equivalent rule set (ERS). This set was constructed to overcome the inability to distinguish between equivalent rules with cross-sectional data. Thus, while local search improves accuracy, it is dependent on the global search performed by the genetic algorithm to resolve the complexity of the networks (S2 Text, S2 Fig).

To test BONITA-RD, simulated data representing 5 samples was generated by BONITA-NP with a rule set and initial states determined by a uniform random distribution. Rules determined by BONITA-RD were then compared with the rule-set used to generate the data.

### BONITA-PA for pathway analysis

BONITA-PA seeks to prioritize nodes that have a large influence over signal flow through the network by assigning node-level impact scores. The impact score, *I*_*g*_, captures the change induced in the network when the node is perturbed. *I*_*g*_ is given by the difference in network state after knockout and knock-in of g:

$$I_{g}=\sum_{i=1}^{d}\sum_{j=1}^{n}((O_{i,j}-Z_{i,j}{)}^{2})$$ (3)

In Eq 3, *j* ranging from 1 to n indicates nodes in the network, *i* ranging from 1 to d indicates samples, *O*_*i*,*j*_ and *Z*_*i*,*j*_ are BONITA-NP outputs when *g* = 0 and *g* = 1 across all iterations, respectively. The comparison of BONITA-PA’s node impact score with graph theoretical measures of node centrality such as degree centrality, eccentricity, shortest-path betweenness, eigenvector centrality and the hubscore and authority scores obtained from the hyperlink-induced topic search algorithm showed no correlation (S4 Text, S4 Fig). The pathway modulation is measured by taking into account impact score and fold difference in the expression across conditions of interest. Specifically, *M*_*p*_ is calculated as follows:

$$M_{p}=\sum_{1}^{n}log\left(I_{g}\right)*\left|log\left(q_{g}\right)\right|*std\left(g\right)$$ (4)

In Eq 4, *q*_*g*_ is the fold difference of *g* and *std*(*g*) is the standard deviation of g across all samples. To calculate the p-value, a distribution of nodes with different impact scores having a range of fold differences is generated. Specifically, the distribution of *M*_*p*_ values is generated by weighting impact scores for a specific pathway’s topology with random fold differences that are re-sampled from the gene expression data. Pathways with at least four genes in the transcriptomic data are considered.

### Simulated data for comparison of pathway analysis methods

To compare BONITA-PA with existing pathway analysis approaches, simulated datasets that resembled biological data were constructed. The data was generated using a negative binomial distribution with gene-wise means and dispersions from existing RNA-seq data [22]. To simulate the modulation of pathways, the expression levels of source nodes were multiplied by log2(-attenuation) where attenuation values were 0.0, 0.5, 1.0, 1.5, and 2.0 as described in Ihnatova et al [2]. This attenuated signal was propagated by BONITA-NP with random rules to simulate inhibition of the entire network mediated by source node inhibition. To test the performance of BONITA- RD, a subset of networks were obtained by searching the KEGG database for Interferon Gamma (IFN-*γ*). These 12 networks were used as test networks since they provide an unbiased set of networks with varying complexity, ranging in size from 13 to 346 nodes. Signal attenuation and propagation was performed 10 times each on the 6 test networks with nodes (genes) in the RNA-seq data, and analysis performed using CLIPPER, CAMERA, and BONITA. CLIPPER determines modulated pathways based on mean and concentration (the inverse of the covariance) matrices. However, for simulation studies, only p-values from CLIPPER comparison of means and not concentration matrices were considered since this improved performance of CLIPPER substantially.

### Pathway analysis of publicly available data

BONITA was rigorously assessed using RNA-seq data. First, BONITA was compared with state-of-the-art pathway analysis approaches using data from the public domain. Second, BONITA’s specificity in detecting disease specific pathway from patient data was investigated. Finally, BONITA’s ability to infer rules from a *de novo* directed network constructed was evaluated.

Comparison of BONITA pathway analysis with CLIPPER and CAMERA was performed using previously published RNA-sequencing data measuring IFN-*γ* signaling modulation in human choriocarcinoma cells [23] and a study representing translational design where peripheral blood mononuclear cells from infants with mild or severe respiratory syncitial virus were assessed by RNA-sequencing [22]. Data was processed using voom [24] for CAMERA or CLIPPER. A set of 37 immunologically relevant KEGG pathways identified in previous studies were utilized because RSV infection and IFN-*γ* stimulation are expected to modulate these pathways [34]. Furthermore, *a priori* selection of biologically relevant pathways reduces the requirement for correction for multiple comparisons. Data from all studies were processed in *R*.

To test whether BONITA identifies disease specific pathways, microarray gene expression data from a set of 36 experiments comparing patients to healthy controls in 15 unique diseases was analyzed [2, 25, 26]. Previously RMA normalized and log2 transformed microarray data was downloaded and was processed to keep probe ID with highest mean expression for each gene symbol. The data was exponentiated with base 2 before running BONITA. The data was retrieved from Gene Expression Omnibus (GEO) using R packages KEGGandMetacoreDzPathwaysGEO and KEGGdzPathwaysGEO [25, 26]. BONITA was applied to KEGG networks associated with each disease in the data set.

Finally, a *de novo* directed network was generated by application of *miic* [27] to RSV data. Miic constructs directed networks by inferring a coexpression network using mutual information. Edges with higher cumulative mutual information than alternative paths are retained and directed based on topological characteristics. For edges that remained bidirectional, two edges, one going in each direction, were inserting before running BONITA-RD.

### Implementation and code availability

BONITA is written entirely in Python and C using genetic algorithms from deap [28]. It has been tested for use with Intel Distribution for Python 2.7. Generation of network representations of rules was performed by modification of previously published code from the Albert Lab [29]. BONITA is designed to be run from the command line by a non-expert user. Code and documentation are available on Github at https://github.com/thakar-Lab/BONITA.

## Results

BONITA- Boolean Omics Network Invariant-Time Analysis- is designed to leverage variance driven by cellular heterogeneity and signal integration for advanced pathway analysis of cross-sectional data, frequently available in translational studies. The accuracy and robustness of BONITA-RD for cross sectional data was assessed using a series of simulation studies, and its application to a *de novo* network. Furthermore, the utility and performance of BONITA-PA is rigorously tested using simulated and publicly available transcriptomic data. Additionally, its performance was also compared to the state-of-art pathway analysis techniques.

### BONITA accuracy in rule determination

BONITA Network Propagation (BONITA-NP) propagates continuous-valued signals across molecular networks with the assumption that bulk transcriptomic measurements are proportional to the number of cells expressing specific genes. The signal propagation depends on the inference of logic rules performed by BONITA rule determination (BONITA-RD), which is optimized to preserve steady states assumed to be represented by the cross-sectional data. The logic rules define integration of signals coming from different genes. To test the performance of BONITA-RD, a subset of networks were obtained by searching the KEGG database for Interferon Gamma (IFN-*γ*). These 12 networks were used as test networks since they provide an unbiased set of networks with varying complexity, ranging in size from 13 to 346 nodes. Simulated data representing cross-sectional measurements were generated for each test network using BONITA-NP and were used as inputs for BONITA-RD. Rules recovered from BONITA-RD were compared to the rules used to generate simulated data. BONITA recovered exact rules used to generate the simulated data with 50% accuracy across test networks. However, multiple logic rules can result in similar cross-sectional outcomes. Hence, the multiple logic rules that produce equivalent cross-sectional outcomes were treated as ‘equivalent’ rule sets (ERS) (see Methods).

ERS facilitated evaluation of accuracy of BONITA-RD within the limits of cross-sectional data. BONITA-RD accuracy reached 87—99% when considering ERS among test networks (Fig 2b). The size of the ERS depicting number of rules in the set varied from just 1 rule to all possible rules (1, 15 and 127 for in-degree 1, 2 and 3, respectively). The size of the ERS was expected to be dependent on signal flow from the shared upstream nodes. Since signal could flow directly or indirectly from such nodes to the node of interest, it is theoretically impossible to distinguish them with cross-sectional data. Consider a network with 3 nodes, A, B, and C, and with edges from A to B, A to C, and from B to C. Under no circumstances will cross-sectional data reveal whether changes in A are propagated to C directly, via B, or both. We wanted to understand whether the size of the ERS was driven by such unsolvable equivalences. To identify these situations, cases where a single node (like A in the network described above) could influence two incoming edges were enumerated. To this end, the sum of intersection between nodes influencing the signal along each pair of incoming edges was calculated as the sum of the shared ancestors between pairs of upstream nodes *U* of the node under investigation. This total ancestor overlap is given by $\sum_{U_{1}\neq U_{2}}\left|A\left(U_{1}\right)\cap A\left(U_{2}\right)\right|$, for all such pairs of upstream nodes *U* where *A* is the set of all ancestors of *U*. The total ancestor overlap and the size of the ERS were highly correlated (Fig 2d, Spearman r = 0.947), demonstrating that alternative paths that were indistinguishable in cross-sectional data lead to unsolvable rules and consequently larger ERS sizes.

The strikingly high accuracy across diverse networks when considering ERS demonstrates that BONITA rule inference can correctly infer rules to the extent they are distinguishable by cross-sectional data. Next, we investigated the impact of network complexity on BONITA-RD. To assess the impact of network size BONITA-RD accuracy was compared with the number of nodes in each test network. Though the test networks have a wide range of sizes, node numbers did not explain differences in accuracy across networks (Fig 2b). To further understand the differences in accuracy, we hypothesized that the accuracy of ERS would be associated with in-degree. Though BONITA-RD restricts the in-degree to 3, decreasing accuracy with increasing original in-degree (Fig 2c, Spearman r = -0.851) was observed. The in-edges are optimized by BONITA-RD, which could compound the inaccuracies introduced by size of the rule space for the nodes with 3 incoming edges. These findings indicate that even when BONITA achieves >80% accuracy, the nodes that are incorrectly inferred have high in-degree in original network.

### Robustness of BONITA to technical noise, sample number, and prior knowledge

**Fig 3:**
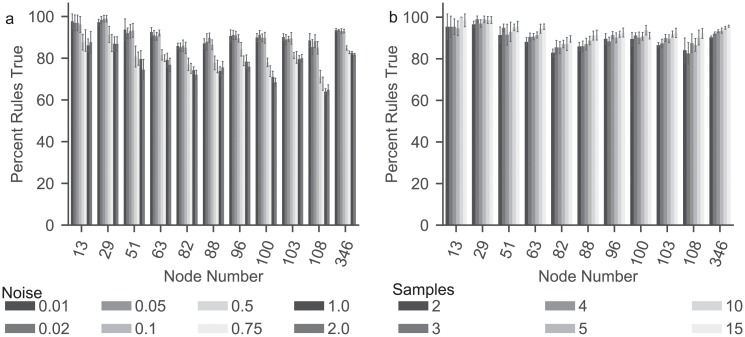
BONITA robustness to noise and sample number is measured on the data simulated using BONITA-NP using test networks for ten trials with various amounts of (a) added noise or (b) samples. ERS (y-axis) for (a) 1% to 200% noise (x-axis) and (b) for 2 to 15 number of samples are reported across the test networks. Error bars represent standard error.

Having established the ability of BONITA-RD to recover rules from large-scale data, we wanted to establish BONITA’s robustness to other important factors in transcriptomic data: sample number and technical noise. Susceptibility of BONITA to technical noise was investigated by adding random noise in the range 1-200% for each node in the network. The BONITA-RD accuracy remains >80% with up to 10% noise in the data (Fig 3a), however accuracy was 65-91% when noise to signal ratio was 50%. Overall, accuracy dropped to 65-88% with addition of 200% noise for larger networks. For sample size analysis, the number of samples were varied from 2 to 15 in the simulated data. Fig 3b shows that accuracy improves from 83-95% with three samples to 91-99% with 15 samples across test networks, but accuracy was preserved to be >80% with just 2 samples. Altogether, these simulations show that BONITA is robust to sample number and technical noise.

**Fig 4:**
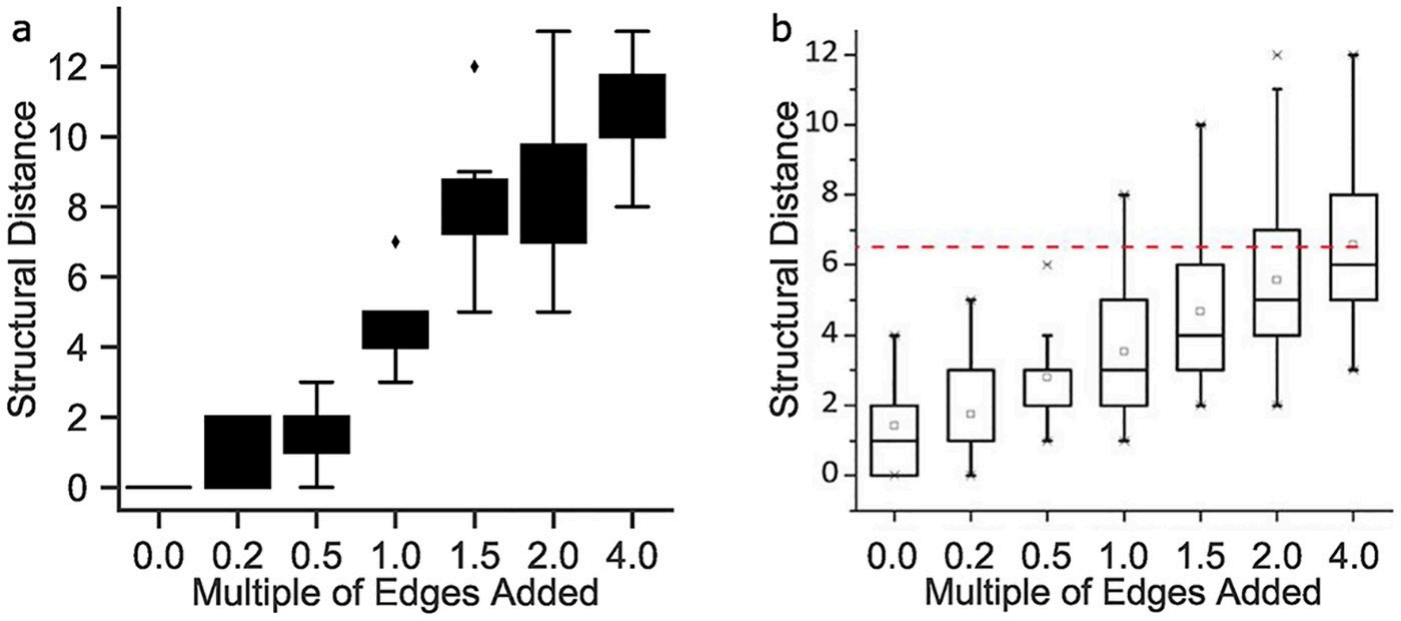
Performance and robustness of BONITA-RD. (a) Boxplots show minimum structural distance among the equivalent rule sets learned by BONITA-RD (y-axis) or (b) structural distance among rules learned by Liu’s method across prior knowledge network [9]. Structural distance is measured as the number of edges that must be added or removed after optimization to obtain the original correct network for addition of false positive edges 0, 0.2, 0.5, 1, 1.5, 2, or 4 times the edge number (x-axis). b) is reproduced from Liu et al. [9] and x-axis labels modified for ease of interpretation.

Typically, pathway topologies available in databases are generalized cases that can lead to false positive edges not relevant to the context of a specific study. Hence, the robustness of BONITA to false positive edges in the prior knowledge network was assessed and compared to the existing algorithm that utilized discrete state modeling [9]. A toy network from [9] was used to generate a dataset and false positive edges were added as multiples of that network’s edge number. The ability of BONITA-RD to retrieve the original network was measured by structural distance. Specifically, structural distance is the number of edges that must be added or removed to obtain the original correct network. BONITA-RD performed substantially better with <0.5 times the number of edges added to the prior knowledge network than Liu’s model, but worse when false-positive edges were greater than the number of edges in the prior knowledge network(Fig 4). Critically, BONITA application to cross-sectional data performs comparably to Liu’s model applied to time course data. Generally, time course data is expected to make rule inference, especially of directional edges, easier. Thus, BONITA-RD performance is robust given the limitations of cross-sectional data in rule inference and relies on reasonably accurate prior knowledge networks.

### Comparison of BONITA-PA accuracy with existing methods

**Fig 5:**
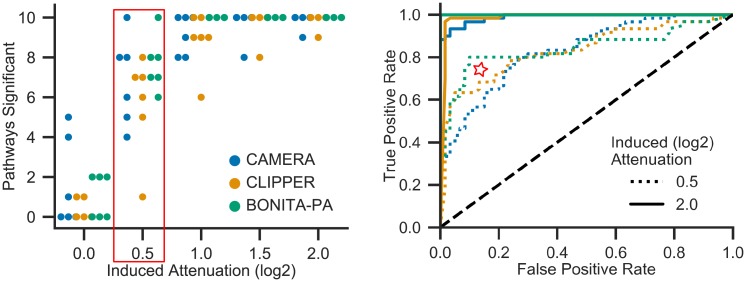
Comparison of BONITA-PA performance with CLIPPER and CAMERA. (a) The number of pathways found to be significant upon attenuation of the source node by *log*_2_ 0.0, 0.5, 1, 1.5, or 2 and (b) Receiver operating characteristic (ROC) curves for *log*_2_ induced attenuation of 0.5 and 2.0 by BONITA-PA (green), CLIPPER (orange) and CAMERA (blue). The total number of pathways tested were 60 for each attenuation using 10 simulated RNA-seq datasets and 6 test networks. ROC curves were constructed by treating −*log*_10_p-values from 0.0 attenuation as one class and −*log*_10_p-values from 0.5 or 2.0 as the other class.

Pathway analysis is the most useful functionality of BONITA-RD. Briefly, nodes of network representing pathway are perturbed *in silico* to measure network-wide changes and calculate a node-level impact score. This impact score is then used to measure pathway-level modulation in the dataset under study. The performance of BONITA-PA was assessed by comparing its output to previously developed topology based pathway analysis method (CLIPPER) and a popular gene-set enrichment method (CAMERA). CLIPPER was chosen since it was the best performing algorithm in a recent comparative analysis of network based pathway analysis techniques [2]. The comparison was performed on the simulated data representing attenuation of pathway source node and downstream events (details in Methods). BONITA was more sensitive than previous methods especially at low levels of source node attenuation (Fig 5, refer to box and star) with the area under the curves (AUCs) 0.842, 0.832 and 0.830 for BONITA, CLIPPER and CAMERA respectively at *log*_2_ attenuation of 0.5. All the methods performed well in detecting the number of pathways for induced attenuation >1 in source nodes (Fig 5b). The performance of all the three was excellent (.99-1.00 AUC) at *log*_2_ attenuation of 2.0. The same results hold when attenuation is not propagated through the downstream nodes of the pathways (S5 Text, S5 Fig). Thus, BONITA-PA outperforms previous state-of-the-art methods at low levels of pathway perturbation. Moreover, even though BONITA-PA performs as well as other methods at high level of signal perturbation, it offers rules for synergy among genes unlike any other methods.

### Comparison of pathway analysis methods for detecting IFN-*γ* signaling perturbations

**Fig 6:**
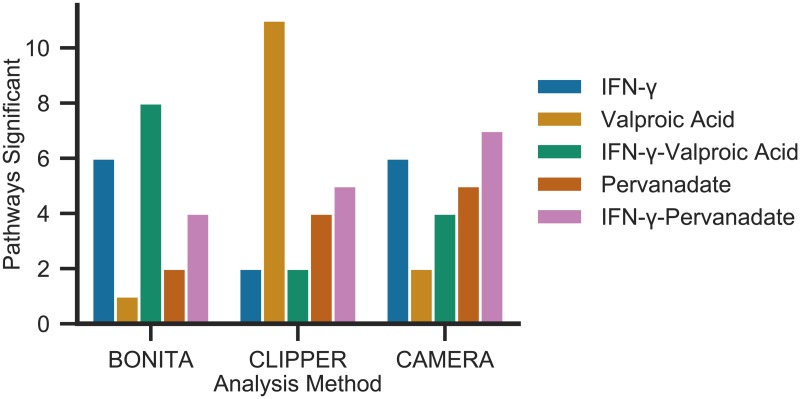
Significant pathways detected upon IFN-*γ* signaling perturbations. Following stimulation of human choriocarcinoma (Jar) cells with IFN-*γ* with or without pervanadate, a protein tyrosine phosphatase inhibitor, or valproic acid, a histone deacetylase (HDAC) inhibitor when compared to untreated cells by BONITA, CLIPPER, and CAMERA. The number of pathways with p<0.05 are shown. Blue represents *IFN* − *γ* alone, yellow valproic acid, green *IFN* − *γ* + valproic acid, red pervanadate and pink *IFN* − *γ* + pervanadate.

BONITA’s excellent performance on simulated data and in modeling pathway modulation calls for verifying its performance in similar experimental setting. RNA-seq data from our previous study investigating Interferon-regulated genes (IRG) following stimulation of human choriocarcinoma (Jar) cells with IFN-*γ* with or without pervanadate, a protein tyrosine phosphatase inhibitor, or valproic acid, a histone deacetylase (HDAC) inhibitor [23] was used. Human choriocarcinoma cells are hypo-responsive to IFN-*γ* stimulation due to impaired activation of the JAK-STAT pathway [30, 31]. This impaired activation could be released by pervanadate and/or valproic acid. We previously showed modulation of certain IRGs by inhibitor alone. Nonetheless, stimulation with both IFN-*γ* and inhibitors did not reveal elicitation of higher number of pathways using existing tools such as CAMERA in [23]. In this study, BONITA, CAMERA, and CLIPPER were used to assess significance of 37 immune pathways in IFN-*γ* treatment with or without inhibitors compared to untreated cells (Fig 6, S1 Table). Both BONITA and CAMERA identified 6 significant pathways (p <0.05) when cells are treated with IFN-*γ* alone. However, BONITA performed better in reproducing IFN-*γ* induced pathways in joint stimulation with inhibitors than CAMERA. Specifically, BONITA revealed activation of two pathways only upon joint treatment of IFN-*γ* and either one of the inhibitors as expected from previous studies [30, 31]. CLIPPER performed poorly in detecting responsiveness to IFN-*γ* treatment and mostly detected pathways when cells were treated with the inhibitors. Thus BONITA’s ability to detect pathways specifically upon joint stimulation is due to the inference of modulation of downstream events by upstream nodes, rather than only detecting downstream modulations.

### Comparison of pathway analysis methods for detecting responses to respiratory syncytial virus infection in infants

Detecting specific pathway signals is a major challenge in genome-wide sequencing studies of human samples due to variation across individuals. Previously, we have measured changes in isolated CD4+ T cells from infants with mild and severe respiratory syncytial virus (RSV) infection by genome-wide mRNA sequencing [22]. It is well understood that the convalescent time point is critical in understanding antigen-specific long term responses required for resolving infections [32–34]. However, our previous work indicates that the changes at the convalescent time point are attenuated. Interestingly, BONITA-PA and CAMERA, but not CLIPPER, identified several pathways as differentially regulated across mild and severe comparison even at the convalescent visit (Table 1).

**Table 1:** Differentially regulated pathways at convalescent visit between infants with mild and severe RSV infection [22].

Pathway/Method:	BON	CLP	CAM
Apoptosis	1.33	0.64	0.42
Cell adhesion molecules (CAMs)	0.10	0.64	1.46
Complement and coagulation cascades	2.56	0.39	0.01
Glycolysis / Gluconeogenesis	0.05	0.51	1.44

−*log*_10_ p-values are shown for analysis of infants with mild vs severe disease at convalescent visit with BONITA (BON), CLIPPER (CLP), and CAMERA (CAM). Significant pathways are highlighted.

**Fig 7:**
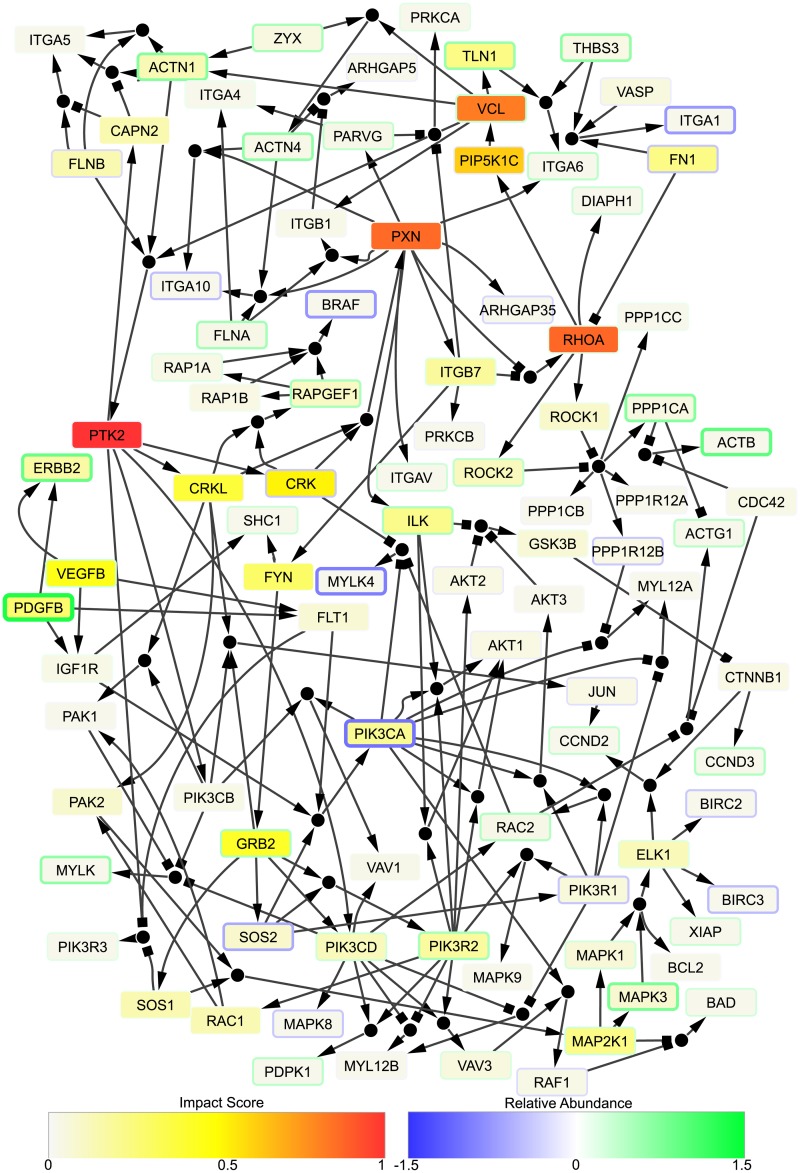
Impact scores, fold differences and rules generated by BONITA-PA for Apoptosis pathway. Small circular nodes indicate ‘and’ rules whereas multiple incoming edges to a rectangular node indicate ‘or’ rule. Colors of the rectangles ranging from white to red indicate low to high impact score. Widths of the rectangles’ outlines and their color ranging from blue to green indicate fold difference (mRNAs) between infants with severe vs mild disease. Blue represents higher expression in mild and green represent higher expression in severe. Impact scores have been divided by the largest impact score in the pathway.

Further, BONITA-PA produces helpful network synthesis, including rules, which can be visualized easily in a network viewer such as Cytoscape, as in Fig 7. This network synthesis was used to investigate the Apoptosis pathway which was detected to be differentially regulated between mild and severe disease by BONITA but not CAMERA or CLIPPER at the convalescent visit. Interestingly, 20 out of 138 nodes obtained high impact score, 5 of which also had >0.5 fold difference between mild and severe. These nodes include many well-known upstream regulators such as PDGFB and PIK3CA [35–37]. Thus, BONITA effectively prioritizes pathway modulation by emphasizing upstream regulators in translational studies.

### Application of BONITA-PA to detect disease specific pathways using patient data

To further test BONITA’s specificity, data from Ihnatova et al. was used [2, 25, 26], which consists of 36 microarray experiments comparing patients with 15 unique diseases to healthy controls. Each of the disease conditions represented in our datasets corresponds to one disease pathway in KEGG. BONITA correctly found corresponding disease pathways to be significant in 22/36 datasets (S2 Table). Ihnatova et al describe that CLIPPER found a comparable number (24/36) to be significant in exactly same comparisons. Further, BONITA has a unique capability to identify nodes with high impact scores, which we hypothesized would be potential drug targets. Drug targets were identified using DrugBank and the targets with indications including the name of the disease pathway (e.g. ‘acute myeloid leukemia’) were retained [38]. Four datasets (3 acute myeloid leukemia and 1 chronic myeloid leukemia) were identified with >1 drug target among high impact nodes designated by BONITA. The enrichment of drug targets among high impact scores was statistically significant (p<0.01, t-test). For example, FLT3, a critical receptor tyrosine kinase mutated in up to 35% of acute myeloid leukemia (AML) cases was found to be one of the three highest impact genes in all three AML datasets. FLT3 is commonly targeted for treatment of AML. Similarly, ABL1, part of the BCR-ABL target of imatinib, an early immunotherapy, had the top impact score in both datasets with chronic myeloid leukemia. Since DrugBank annotations might not be complete, the top 2 impact score nodes in each disease network with p<0.05 in BONITA-PA were manually queried as targets of drugs either under development or approved. This revealed that high impact nodes in each network, except those in Alzheimer disease network, were either targeted by or were the ligand of a receptor targeted by an approved or under development drug (S2 Table). In Alzheimer disease, there are no mechanistic drugs. However, 1/4 datasets revealed TNFRSF1A, the *TNF* − *α* receptor, candidacy of which is supported by previous studies [39–41] (S2 Table). Interestingly, ADRB1, the *β* − 1-adrenergic receptor was the second highest impact gene for dilated cardiomyopathy, which is often treated with beta-blockers targeting the adrenergic receptor (S2 Table). Thus, not only is BONITA-PA able to detect differences in relevant disease networks between patients and healthy control subjects, but it is also highly effective in identifying promising drug target genes. BONITA connects upstream differences with downstream effects, identifying true cascades depicted by the pathway topology that are highly modulated in comparison of interest. Taken together, these results show the effectiveness of BONITA-PA in prioritizing pathways for further experimental studies following genome-wide transcriptional profiling.

### Rule determination in *de novo* inferred gene-regulatory network

One of the applications of BONITA is to define co-operativity in networks inferred from the data. Mutual information-based inductive causation (*miic*) [27] was used to generate a directed network using RSV infection dataset described in the previous section. BONITA was run to obtain logic rules and impact scores. BONITA predicts that absence of TAXBP1, a gene known to participate in restricting antiviral signaling and YPEL5, a gene involved in cell cycle progression leads to activation of TRAF3IP3, which is supported by previous studies [42, 43]. Finally, MYC and SP100 are hypothesized to activate MX1 together. This is particularly interesting since MX1 is a nuclear factor known to recruit SP100 and involved in antiviral response [44]. Thus, in addition to application of BONITA for pathway analysis, it can have high utility in *de novo* hypothesis generation.

## Discussion

BONITA is, to our knowledge, the first ever attempt to use discrete-state modeling for pathway analysis and builds upon decades of work to calculate node impacts in Boolean networks, Probabilistic Boolean Networks and fuzzy logic networks [45]. BONITA uses cross-sectional data along with network topology to find node specific impact scores. The impact scores consider both the learned rules defining synergy among genes and the condition-specific distribution of expression values. Since BONITA-RD recovers rules, *in silico* generation of hypotheses for downstream effects of node perturbations (knockout, knockdown, knock-in) are trivial. In this way, BONITA offers an extension to pathway analysis that no other approach affords.

BONITA-RD is a novel approach to rule determination for cross-sectional data that offers significant advantages over previous algorithms. Existing software can solve the key problem of Boolean rule determination for large-scale omics datasets by use of genetic [9, 14], linear, or nonlinear programming algorithms [12, 46]. These implementations, however, require time-course data which is infrequent in translational studies, limiting their usability. Indeed, BONITA-RD shows comparable robustness and accuracy to the previous algorithms which were solely developed for time-series data [9]. Moreover, these methods require either strictly Boolean or fuzzy values, missing the cell-based variability arising between on and off states. These limitations have hampered the adoption of discrete state modeling in the analysis of omics data.

Approaches to solving networks for cross-sectional data must apply more general optimization solutions because there are no explicit transitions available. Though efficient, genetic algorithms often do not find the best configuration when combinatorial possibilities are high, i.e., when network topology is complex. BONITA-RD combines an exhaustive node-wise local search with a genetic algorithm and achieves high accuracy in determining rules from simulated data. While local search improves accuracy, it is dependent on an initial global search to resolve the complexity of the networks. BONITA-RD is robust to inaccuracies in prior knowledge networks, noise, and number of samples. This optimization happens relatively rapidly within the genetic algorithm (Fig 2a) and the current settings are sufficient for significantly larger and more complex networks than are studied in this report.

In addition to making rule determination possible from cross-sectional data, the BONITA-NP algorithm accounts for cellular heterogeneity by explicitly modeling a population of cells with a distribution of on/off starting states, rather than from varying levels of expression in each as modeled by fuzzy models. Not all genes vary in a switch-like manner by cell, but those that vary in a fuzzy manner will be implicitly modeled (with similar accuracy) in a pseudo-switch-like manner, because the internal direction of gene activation will remain the same. As expected, this model outperforms purely Boolean approaches in terms of error across pathways (S2 Text, S2 Fig). In the future, BONITA can be extended to group the cells into subpopulations and to derive the estimation of transition across cell states/ subpopulations [20]. However, such inferences from the bulk transcriptomic data are non-trivial and newer techniques such as single-cell transcriptomics would facilitate the development.

Rigorous testing of rule inference is a difficult problem. The DREAM challenge provides rigorously validated time-series data sets for evaluation of novel algorithms; however, no such test sets exist for rule inference from cross-sectional data. Hence, a well-controlled study from our collaborator Dr. Shawn Murphy was used to validate BONITA [30, 31] (Fig 6). BONITA’s ability to identify pathways specifically in case of joint-stimulation with IFN-*γ*-valproic acid and IFN-*γ*-pervanadate corroborates with multiple previously published studies [30, 31]. Previous methods could not distinguish increased immune signaling with co-treatment of IFN-*γ* and valproic acid or pervanadate, demonstrating that BONITA can extract useful biological information. Furthermore, rigorous testing of accuracy and robustness to noise, errors in the prior knowledge network and number of samples demonstrates the effectiveness of BONITA-RD in learning rules from cross-sectional data.

Interestingly, mutually exclusive pathways were identified by CAMERA and BONITA at the convalescent visit after RSV infection but no pathways identified by CLIPPER. The pathways were most likely mutually exclusive because BONITA has higher sensitivity to detect pathways with upstream changes that are linked to downstream variation whereas CAMERA will detect changes in downstream genes as observed in the glycolysis pathway even in the absence of corresponding changes in upstream regulators of a pathway. Non-signaling networks like glycolysis may have unclear signal flows, as described in the Methods, or may contain many loops. In these cases, BONITA’s performance will be similar to that of other gene-set analysis methods instead of the enhanced performance observed on signaling networks when causation of downstream events can be linked to the upstream changes.

**Fig 8:**
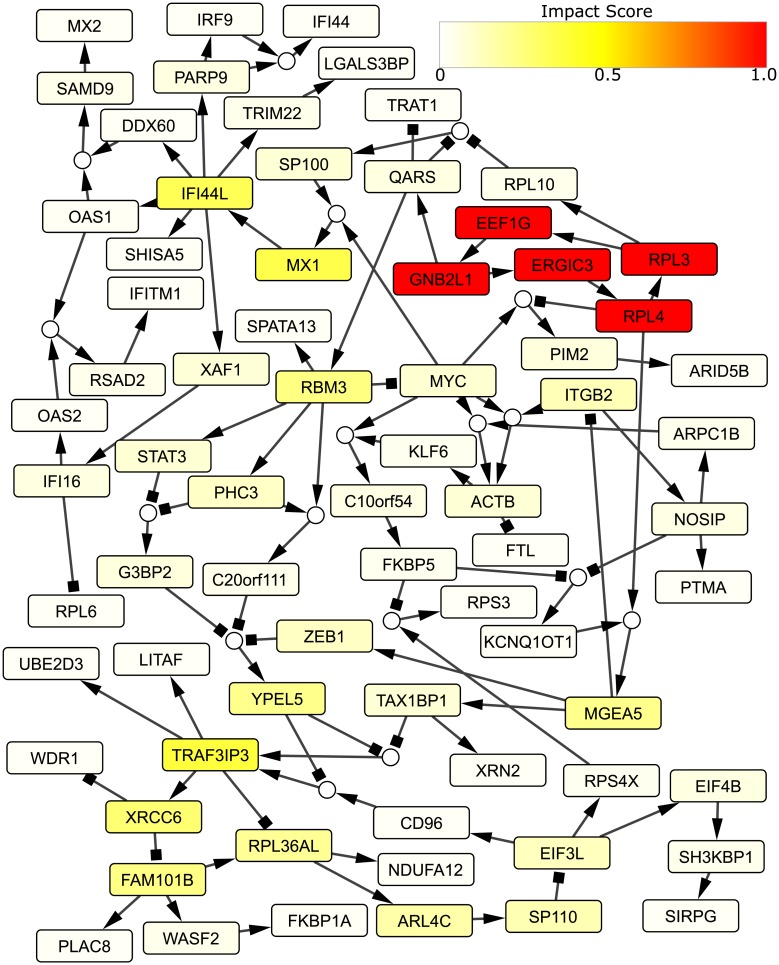
Rules generated by BONITA-RD for *de novo* network. BONITA-RD and impact score calculation were applied to a network generated by mutual information-based inductive causation (miic) [27]. Small circular nodes indicate ‘and’ rules whereas multiple incoming edges to a rectangular node indicate ‘or’ rule. Colors of the rectangles ranging from white to red indicate low to high impact score. Impact scores have been divided by the largest impact score in the pathway.

BONITA-PA explicitly provides increased impact to upstream nodes in the context of downstream nodes. This quantitative prioritization of upstream signaling and relative modulation highlights nodes and interactions that make pathways most interesting for further exploration. The utility of such an approach is underscored by the effectiveness of BONITA impact scores in identifying drug targets. Thus, BONITA provides a unique perspective and new capabilities to maximize the utility of transcriptomics experiments in guiding future studies. Further, BONITA can be applied to *de novo* inferred networks (Fig 8), extending its use to create a complete platform to capture network of interactions from transcriptomic data. Finally, in addition to the applications described here, accurate models of all-or-none behavior in heterogeneous populations like those described by BONITA-NP have broad applicability for diverse types of molecular networks. Thus BONITA offers a novel tool for mechanistic interpretation of transcriptomic data.

In conclusion, BONITA introduces a new, useful, and conceptually elegant approach to considering variance in transcriptomic data. BONITA is theoretically applicable to any directed network, including *de novo* inferred regulatory networks. Future releases of the BONITA software will include interfaces to other pathway databases. Further developments in transcriptomics technology and *de novo* assembly of directed networks from these rich data sets will enhance the applicability and usefulness of the BONITA approach.

## Supporting information

S1 Table Results summary of comparison of BONITA to CAMERA and CLIPPER for detection of *IFN* − *γ* signaling perturbations. −*log*10 p-values from pathway analysis of human choriocarcinoma cells after treatment with/ without IFN-*γ*, phosphatase inhibitor (pervanadate) and histone deacetylase inhibitor (valproic acid).
(XLSX)

S2 Table BONITA results summary for human disease data set. Results of BONITA simulation across human disease data sets (p-values) along with the number of drug targets and, if applicable, p value of t test of impact scores between drug targets and non-drug target nodes.
(XLSX)

S1 Fig Distribution of median rule number from ERS obtained from 25 trials for each test network of BONITA-RD from simulated data using random rules as in main text Fig 2. (EPS)

S2 Fig Performance of BONITA-RD with rescaling or binarization methods. BONITA-RD was optimized using the RSV infection data [22] transformed using continuous rescaling (top 3) or binarization (bottom 5) methods. The mean squared error (MSE) between the transformed data and the values estimated by BONITA across 3 replicates and all IFNG networks are plotted along with standard error represented by error bars.
(EPS)

S3 Fig Histogram of length of longest shortest path between all pairs of nodes in the test networks. (EPS)

S4 Fig BONITA node impact score shows low correlation to node centrality measures. Values in the labeled cells represent the Pearson correlation coefficient. Colors also represent Pearson correlation coefficient, ranging from -1 (dark blue) to 1 (dark red).
(EPS)

S5 Fig Comparison of BONITA-PA performance with CLIPPER and CAMERA in simulated data. (a) The number of pathways out of ten found to be significant in simulated RNA-seq data with source nodes of 10 random data sets each of 6 test networks attenuated by *log*_2_ 0.0, 0.5, 1, 1.5, or 2 without propagation to downstream nodes. (b) Receiver operating characteristic (ROC) curves for *log*_2_ induced attenuation of 0.5 and 2.0 without propagation to downstream nodes. Receiver operating characteristic (ROC) curves were constructed by treating −*log*_10_ p-values from 0.0 attenuation as one class and −*log*_10_ p-values from 0.5 or 2.0 as the other class. Green represents BONITA-PA, orange represents CLIPPER and blue represents CAMERA in both a and b.
(EPS)

S1 Text Characterization of equivalent rule sets. (PDF)

S2 Text Comparison of methods for [0, 1] transformation and binarization. (PDF)

S3 Text Determination of number of simulation steps in BONITA-NP for KEGG networks. (PDF)

S4 Text BONITA node impact score does not correlate with node centrality measures. (PDF)

S5 Text Comparison of BONITA-PA performance with CLIPPER and CAMERA in simulated data without network propagation. (PDF)
