# Supplementary material for: Executable pathway analysis using ensemble discrete-state modeling for large-scale data
Source: PLoS Comput Biol. 2019 Sep 3;15(9):e1007317. doi: 10.1371/journal.pcbi.1007317 (PMC6743792; doi:10.1371/journal.pcbi.1007317)
Supplement: S1 Text — (PDF) [file pcbi.1007317.s008.pdf]

Text S1

Characterization of Equivalent Rule Sets

The number of possible Boolean functions for a network of given topology is extremely large [1]; however, it is possible to restrict the search space for BONITA-RD by restricting the candidate functions to the desired mathematical form. Zhou et. al. [2] have shown that restricting the search space to sign-compatible functions results in ordered, directional network dynamics. As described in the Materials and Methods, we exploit the edge annotations in the directional KEGG networks to assign “activation” or “inhibition”, and thus infer only functions that are positively or negatively unate respectively (i.e. we consider only sign-compatible functions). This restriction significantly reduces the space that BONITA-RD must search. We demonstrate in Fig 2, panel d of the main text that BONITA-RD successfully narrows down the size of the potential ERS for a given node except when there is a high degree of overlap between the ancestors of the inputs to that node. Fig **S1** shows the distribution of the rule number metric defined in [2], for the set of networks and their corresponding ERS across 25 trials described in Fig 2. Briefly, the rule number of a Boolean function is the number of components separated by an ‘OR’ in the disjunctive normal form of the Boolean function. Since we restrict nodes to 3 incoming edges, the maximum value of rule number is 7. We note that BONITA-RD appears to infer rules in an unbiased manner (mean rule number=3.72). This implies that the rules are in accordance with the characteristics of a rugged Boolean network landscape i.e. a landscape with more difficult transitions between states [2]. For the ERS of a given node, rules with both high and low rule number are represented (even though the size of the ERS tends to be small, see Fig 2d in the main text). The choice of which rules to select for future investigations is left to the discretion of the end-user. A parsimonious rule set implies a flat Boolean network landscape, and may be useful for simulations intended to visit all possible attractors. We have added a command line utility for producing such plots from BONITA-PA for advanced users.

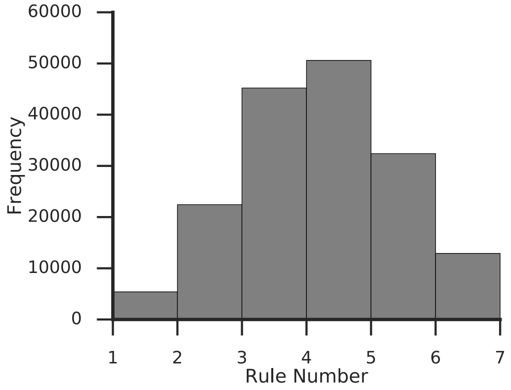

Fig S1. **Distribution of median rule number from ERS** obtained from 25 trials for each test network of BONITA-RD from simulated data using random rules as in main text Fig. 2

References

[1] Henry, A. and Moneger, F. and Samal, A. and Martin, O. C. Network function shapes network structure: the case of the Arabidopsis flower organ specification genetic network Mol Biosyst 2013;9:7:1726-35 doi: 10.1039/c3mb25562j

[2] Zhou J, Samal A, d'Hérouël A, Price N, Huang S. Relative Stability of Network States in Boolean Network Models of Gene Regulation in Development.. Biosystems. 2016;142–143:15–24. doi:https://doi.org/10.1016/J.BIOSYSTEMS.2016.03.002.
