# Supplementary material for: Executable pathway analysis using ensemble discrete-state modeling for large-scale data
Source: PLoS Comput Biol. 2019 Sep 3;15(9):e1007317. doi: 10.1371/journal.pcbi.1007317 (PMC6743792; doi:10.1371/journal.pcbi.1007317)
Supplement: S2 Text — (PDF) [file pcbi.1007317.s009.pdf]

Text S2

Comparison of methods for [0,1] transformation and binarization

To test the performance of BONITA-RD with different methods for data transformation in $[0, 1]$ domain i.e. calculating active cell proportions from real data, the following methods were compared using data described in [8]- feature rescaling, fuzzy c-means clustering [1, 2] and division by the maximum element. The mean square errors were evaluated (Fig S2) to find that the division by the maximum element produced the lowest average error across networks (Fig S2). In addition, the following binarization methods were compared - BASC-A, BASC-B [3], k-means clustering [4], edge detection [5] and binarization by the scan statistic [6] that have been implemented in BoolNet [6] and BiTriNa [7]. As expected, binarization methods produce higher node-wise errors than continuous rescaling methods (Fig S2) due to loss of information during discretization at the cut-off point. Thus, division by the maximum more closely recapitulates data compared to the other continuous transforms or, as expected, conventional Boolean methods and was used in this manuscript.

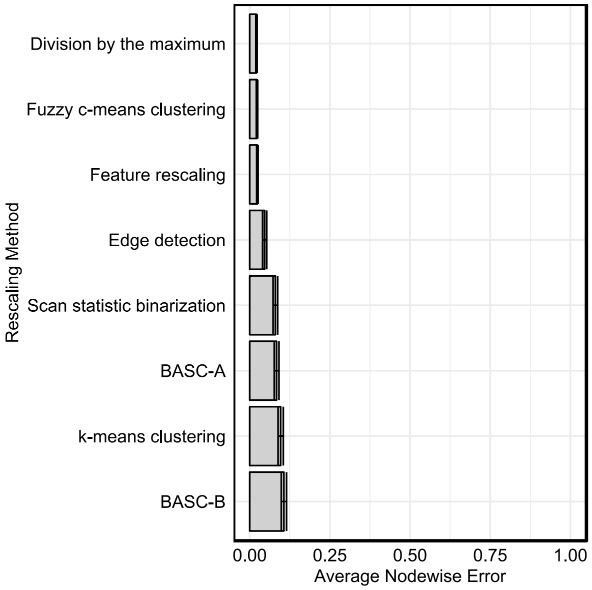

Fig S2. Performance of BONITA-RD with rescaling or binarization methods. BONITA-RD was optimized using the RSV infection data [8] transformed using continuous rescaling (top 3) or binarization (bottom 5) methods. The mean squared error (MSE) between the transformed data and the values estimated by BONITA across 3 replicates and all IFNG networks are plotted along with standard error represented by error bars.

References

[1] Dembele D, Kastner P. Fuzzy C-means method for clustering microarray data. Bioinformatics. 2003;19(8):973–980. doi:10.1093/bioinformatics/btg119.

[2] Khan A, Katanic D, Thakar J. Meta-analysis of cell- specific transcriptomic data using fuzzy c-means clustering discovers versatile viral responsive genes. BMC Bioinformatics. 2017;18(1):295.

[3] Hopfensitz M, Mussel C, Wawra C, Maucher M, Kuhl M, Neumann H, et al. Multiscale Binarization of Gene Expression Data for Reconstructing Boolean Networks. IEEE/ACM Transactions on Computational Biology and Bioinformatics. 2012;9(2):487–498. doi:10.1109/tcbb.2011.62.

[4] MacQueen J. Some methods for classification and analysis of multivariate observations. In: Proceedings of the Fifth Berkeley Symposium on Mathematical Statistics and Probability, Volume 1: Statistics. Berkeley, Calif.: University of California Press; 1967. p. 281–297. Available from: `https://projecteuclid.org/euclid.bsmsp/1200512992`.

[5] Shmulevich I, Dougherty ER, Zhang W. Control of Stationary Behavior in Probabilistic Boolean Networks by Means of Structural Invervention. Journal of Biological Systems. 2002;10(04):431–445. doi:10.1142/S0218339002000706.

[6] Müssel C, Hopfensitz M, Kestler HA. BoolNet—an R package for generation, reconstruction and analysis of Boolean networks. Bioinformatics. 2010;26(10):1378–1380. doi:10.1093/bioinformatics/btq124.

[7] Müssel C, Schmid F, Blätte TJ, Hopfensitz M, Lausser L, Kestler HA. BiTrinA—multiscale binarization and trinarization with quality analysis. Bioinformatics. 2015;32(3):465–468. doi:10.1093/bioinformatics/btv591.

[8] Mariani TJ, Qiu X, Chu C, Wang L, Thakar J, Holden-Wiltse J, et al. Association of Dynamic Changes in the CD4 T-Cell Transcriptome With Disease Severity During Primary Respiratory Syncytial Virus Infection in Young Infants. The Journal of Infectious Diseases. 2017;216(8):1027–1037. doi:10.1093/infdis/jix400.
