# Supplementary material for: Executable pathway analysis using ensemble discrete-state modeling for large-scale data
Source: PLoS Comput Biol. 2019 Sep 3;15(9):e1007317. doi: 10.1371/journal.pcbi.1007317 (PMC6743792; doi:10.1371/journal.pcbi.1007317)
Supplement: S3 Text — (PDF) [file pcbi.1007317.s010.pdf]

Text S3

Determination of number of simulation steps in BONITA-NP for KEGG networks

To investigate the adequate number of simulation steps for BONITA-NP across diverse networks, all KEGG networks (186) from MSigDb were used [1]. The number of maximum simulation steps can be approximated by the maximum distance a signal could travel between any pair of nodes. Hence we evaluated the length of the longest shortest path between any two nodes. The maximum value of this statistic is 17 (Fig S3), indicating that the number of simulation steps (100) used in BONITA-NP is approximately 7 times greater than required for KEGG networks.

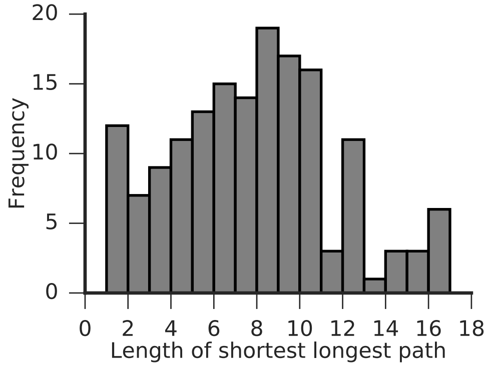

Fig S3. Histogram of length of longest shortest path between all pairs of nodes in KEGG networks

References

[1] Liberzon, A.,Subramanian, A., Pinchback, R.,Thorvaldsdottir, H., Tamayo, P. and Mesirov, JP. Molecular signatures database (MSigDB) 3.0 Bioinformatics 2011; 27(12):1739-1740 doi:10.1093/bioinformatics/btr260
