# Supplementary material for: Executable pathway analysis using ensemble discrete-state modeling for large-scale data
Source: PLoS Comput Biol. 2019 Sep 3;15(9):e1007317. doi: 10.1371/journal.pcbi.1007317 (PMC6743792; doi:10.1371/journal.pcbi.1007317)
Supplement: S4 Text — (PDF) [file pcbi.1007317.s011.pdf]

Text S4

BONITA node impact score does not correlate with node centrality measures

BONITA calculates a node impact score (Eqn 4) measuring its importance in signal flow through a network by calculating the difference in overall network state when that node is knocked in or out. To investigate whether impact scores are associated with topological characteristics [1], we selected 6 representative measures of node centrality: degree centrality, eccentricity, shortest-path betweenness, and eigenvector centrality in addition to the hub-score and authority scores obtained from the hyperlink-induced topic search algorithm. The centrality measures were compared by Pearson correlation [2] to the impact scores calculated upon optimization of BONITA-RD for RSV infections dataset (see main text, Materials and Methods). Weak correlation of impact scores with the selected centrality measures (see Fig S4) indicates that the node impact score provides information orthogonal to that of traditional graph theoretic metrics. This was expected since BONITA impact score is designed to prioritize upstream nodes including source nodes the most whereas traditional centrality metrics prioritize nodes in the middle.

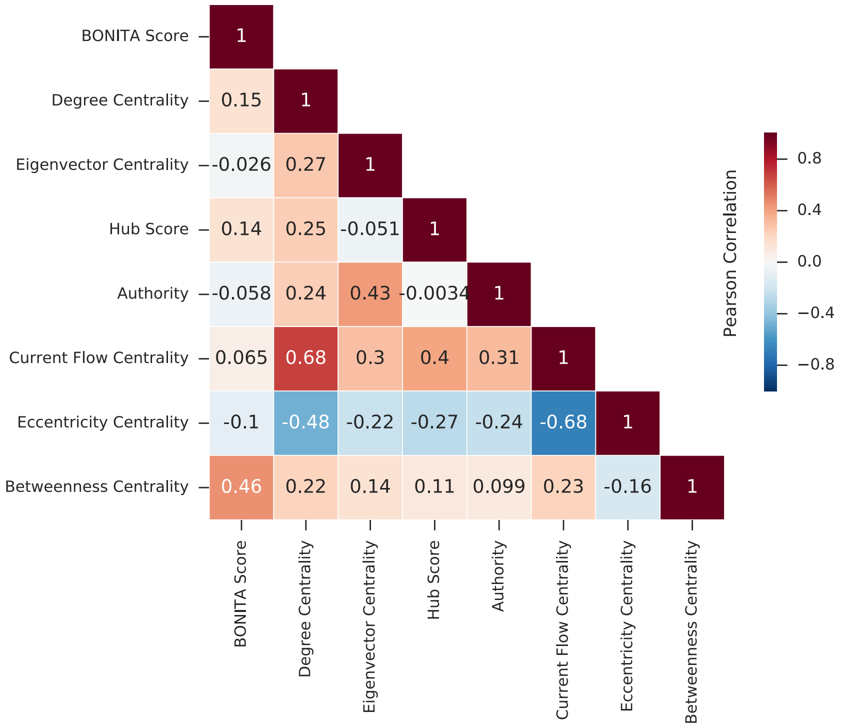

Fig S4. BONITA node impact score shows low correlation to node centrality measures. Values in the labeled cells represent the Pearson correlation coefficient. Colors also represent Pearson correlation coefficient, ranging from -1 (dark blue) to 1 (dark red).

References

[1] Mahdieh G., Hossein S., Faezeh T., Maseud R. and Ali M. Centrality Measures in Biological Networks Current Bioinformatics. 2014;9(4):426-441 http://dx.doi.org/10.2174/157489361130866600l3 2014

[2] Koschützki, D., and Schreiber, F. Centrality Analysis Methods for Biological Networks and Their Application to Gene Regulatory Networks Gene Regulation and Systems Biology. 2008;2:1177-6250 https://doi.org/10.4137/GRSB.S702
