# Supplementary material for: Executable pathway analysis using ensemble discrete-state modeling for large-scale data
Source: PLoS Comput Biol. 2019 Sep 3;15(9):e1007317. doi: 10.1371/journal.pcbi.1007317 (PMC6743792; doi:10.1371/journal.pcbi.1007317)
Supplement: S5 Text — (PDF) [file pcbi.1007317.s012.pdf]

Text S5

Comparison of BONITA-PA performance with CLIPPER and CAMERA in simulated data without network propagation

To further assess comparison of BONITA-PA with existing pathway analysis approaches, we replicated Fig 5 of the main text using the same initial simulated data. Pathway modulation was similarly accomplished by multiplying the abundance levels of source nodes by log2(-attenuation) where attenuation values were 0.0, 0.5, 1.0, 1.5, and 2.0 as described in Ihnatova et al [1]. Unlike in Figure 5, this attenuated signal was not propagated by BONITA-NP. Pathway analysis was performed ten times each on 6 test pathways following above simulation procedure using CLIPPER, CAMERA and BONITA as in main text (see methods). Results shown in the main text are recapitulated with AUC of 0.839, 0.723, and 0.810 for BONITA, CLIPPER and CAMERA respectively at log_2 attenuation of 0.5 (Fig S5 b). Thus, BONITA-PA outperforms previous state-of-the-art methods at low levels of pathway perturbation even when BONITA-NP is not used to generate simulated data.

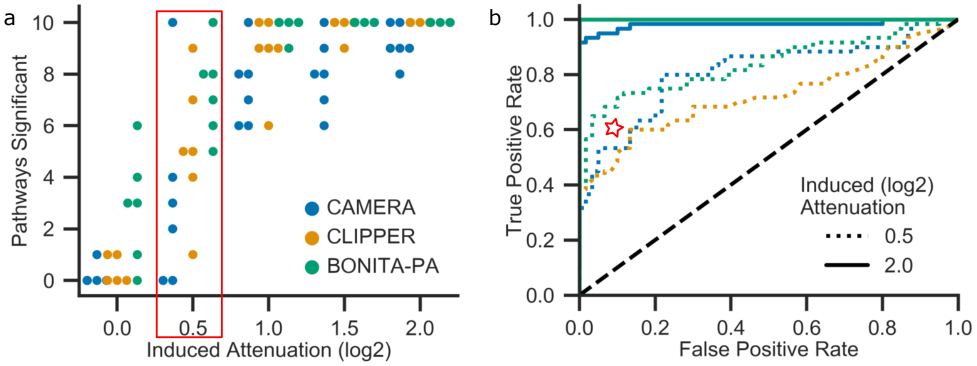

Fig S5. Comparison of BONITA-PA performance with CLIPPER and CAMERA in simulated data. (a) The number of pathways out of ten found to be significant in simulated RNA-seq data with source nodes of 10 random data sets each of 6 test networks attenuated by log_2 0.0, 0.5, 1, 1.5, or 2 without propagation to downstream nodes. (b) Receiver operating characteristic (ROC) curves for log_2 induced attenuation of 0.5 and 2.0 without propagation to downstream nodes. Receiver operating characteristic (ROC) curves were constructed by treating $-log_{10}$ p-values from 0.0 attenuation as one class and $-log_{10}$ p-values from 0.5 or 2.0 as the other class. Green represents BONITA-PA, orange represents CLIPPER and blue represents CAMERA in both a and b.

References

[1] Ihnatova I, Popovici V, Budinska E. A critical comparison of topology-based pathway analysis methods. PLOS ONE. 2018;13(1):e0191154. doi:10.1371/journal.pone.0191154.
